# Quantifying malaria endemicity in Ethiopia through combined application of classical methods and enzyme-linked immunosorbent assay: an initial step for countries with low transmission initiating elimination programme

**DOI:** 10.1186/s12936-018-2282-9

**Published:** 2018-04-04

**Authors:** Zewdie Birhanu, Yemane Ye-ebiyo Yihdego, Delenasaw Yewhalaw

**Affiliations:** 1 Department of Health Education and Behavioral Sciences, Jimma University, Jimma, Ethiopia; 2 Abt Associates, Africa Indoor Residual Spraying, Accra, Ghana; 3 Department of Medical Laboratory Sciences and Pathology, Jimma University, Jimma, Ethiopia; 4 Tropical and Infectious Diseases Research Center, Jimma University, Jimma, Ethiopia

**Keywords:** Malaria, Endemicity, Splenomegaly, Malaria antibody, Malaria serology, Malaria EIA, Malaria parasitaemia, Ethiopia

## Abstract

**Background:**

In the context of reduced transmission of malaria, it is essential to re-evaluate and determine the level of transmission as it guides re-orientation of control measures which is appropriate to local disease epidemiology. However, little is known about level of malaria transmission in Ethiopia. The present study aimed to investigate the level of malaria transmission through combined application of classical methods and enzyme-linked immunosorbent assay (EIA) in low transmission settings of Ethiopia.

**Methods:**

This study was conducted in June 2016 on 763 apparently healthy children 2–9 years of age. Children were recruited from ten sites representing different malaria transmission settings in Ethiopia. Splenomegaly rate, infection rate and EIA antibody test were used to determine endemicity. The data were analysed using SPSS 21.0 and Stata 12.0.

**Results:**

The overall prevalence of malaria parasitaemia was 2.49% (95% CI 1.38–3.59) and 2.36% (95% CI 1.28–3.44) as detected using rapid diagnostic test and microscopy, respectively. *Plasmodium falciparum* accounted for 62.63% of the infections. The prevalence of parasitaemia significantly varied by altitude and localities; the highest (5.8%) in areas below 1500 m above sea level. Overall, splenomegaly rate was 1.70% (95% CI 0.78–0.2.66%), making the overall malaria transmission hypoendemic. Infection rate was higher among males (2.7%), but rate of splenomegaly was higher in females. Incongruent with spleen rate and parasitaemia, EIA showed a higher level of cumulative exposure to malaria with spatially localized and highly heterogeneous transmission. Overall, 126 (18.75%, 95% CI 15.79–21.71) of the children were positive for total malaria antibodies with significant variations with altitude, age and sex; the higher in areas of < 1500 m asl (25.8%), children ≥ 5 years (22.1%) and among males (20.9%).

**Conclusions:**

Splenomegaly and parasitaemia are not good measures to show variations in the levels of malaria transmission in reduced and/or low endemic settings. The malaria antibody (i.e. serological) test seems to be a good measure of malaria endemicity showing greater degree of heterogeneity and localized risk of transmission. Thus, malaria elimination efforts need to be supported with serological indicators to identify patterns of foci of transmission to set priorities for interventions.

## Background

Despite remarkable achievements, the human toll of malaria and the global risk it still poses remains unacceptably high [1, 2]. In 2015, nearly half of the world’s population was living in areas with ongoing malaria transmission. During the same year, 214 million people caught malaria, and it caused approximately 438,000 deaths, globally. The African region contributed about 88 and 90% of the world’s malaria cases and deaths, respectively [1–4]. In most malaria endemic countries, there is an ambitious target for reduction of malaria burden and many countries are moving towards elimination [1, 2]. In order to accelerate the progress towards global malaria eradication programme, it is essential to hasten the current progress by reducing morbidity and mortality in all endemic countries by identifying context-specific approaches and interventions that fit to the local disease epidemiology [2–4]. Thus, it is a critical juncture for malaria endemic countries and territories to make the response to malaria more systematic, evidence-based focused and context-driven interventions [2].

In low transmission areas, many people who are infected with malaria parasites remain asymptomatic and, therefore, undetected by the health system. Such individuals make a significant contribution to sustain malaria transmission in the community [2, 5–11]. If future malaria control and elimination programmes are to succeed, they should take into account the heterogeneity of malaria transmission and should be supported by evidence that guides re-orientation of control measures [2–4]. Thus, the prospects of malaria elimination require a proper understanding of the local disease endemicity, which could guide re-orientation of planning control measures concordant to the local transmission pattern [2].

Despite promising progresses made to date [3–5], malaria remains one of the top ranking health problems in Ethiopia. In 2015, over two million malaria cases (confirmed plus clinical) were recorded, responsible for 662 deaths [12, 13]. Of these total cases, *Plasmodium falciparum* accounted for 63.7%, and the remaining were due to *Plasmodium vivax* [13]. In Ethiopia, over 60% of the population lives in areas at risk of malaria infection as of 2014 [14, 15]. In the country, populations living in areas below 2000 m above sea level are considered to be at risk of malaria. Malaria transmission intensity is generally unstable and seasonal [14, 15]. In most part of the country, the peak period of malaria transmission occurs between September and December following the main rainy season (June–September). A second minor transmission period occurs from April to June, following a short rainy season of February to March. Since peak malaria transmission often coincides with the planting and harvesting season, there is a heavy economic burden in Ethiopia. Ethiopia has set targets and initiates malaria elimination programme to see malaria free Ethiopia by 2030 [14, 15]. In this regard, it is aimed to reduce malaria death to 0.6 per 100,000 population at risk, reduce prevalence of malaria parasite to less than one percent; and eliminate malaria from 200 districts with low transmission by 2020 [14, 15].

In Ethiopia, malaria prevention and control strategies have substantially remained unchanged over the past decade [14, 15]. This entails the need to re-evaluate malaria endemicity in the country to develop strategies to sustain control and support elimination programme. As malaria transmission declines, in countries like Ethiopia, the heterogeneity in incidence and transmission may likely increase requiring inventions tailored to local contexts [2–5, 7–11]. Understanding local malaria endemicity could help to optimize malaria responses; guides the choice of appropriate intervention strategy; helps to re-orient control activities, and is necessary to reinvigorate political commitment to accelerate efforts towards elimination. However, little is documented regarding endemicity of malaria in Ethiopia and most importantly, given the reduced malaria transmission a in the country, there is strong need to generate evidence on the current level of malaria in the country. Therefore, this study investigated malaria endemicity in Ethiopia through combined use of classical measures (parasite rate and splenomegaly rate) and enzyme-linked immunosorbent assay (EIA) antibody test.

## Methods

### Study settings

**Fig. 1:**
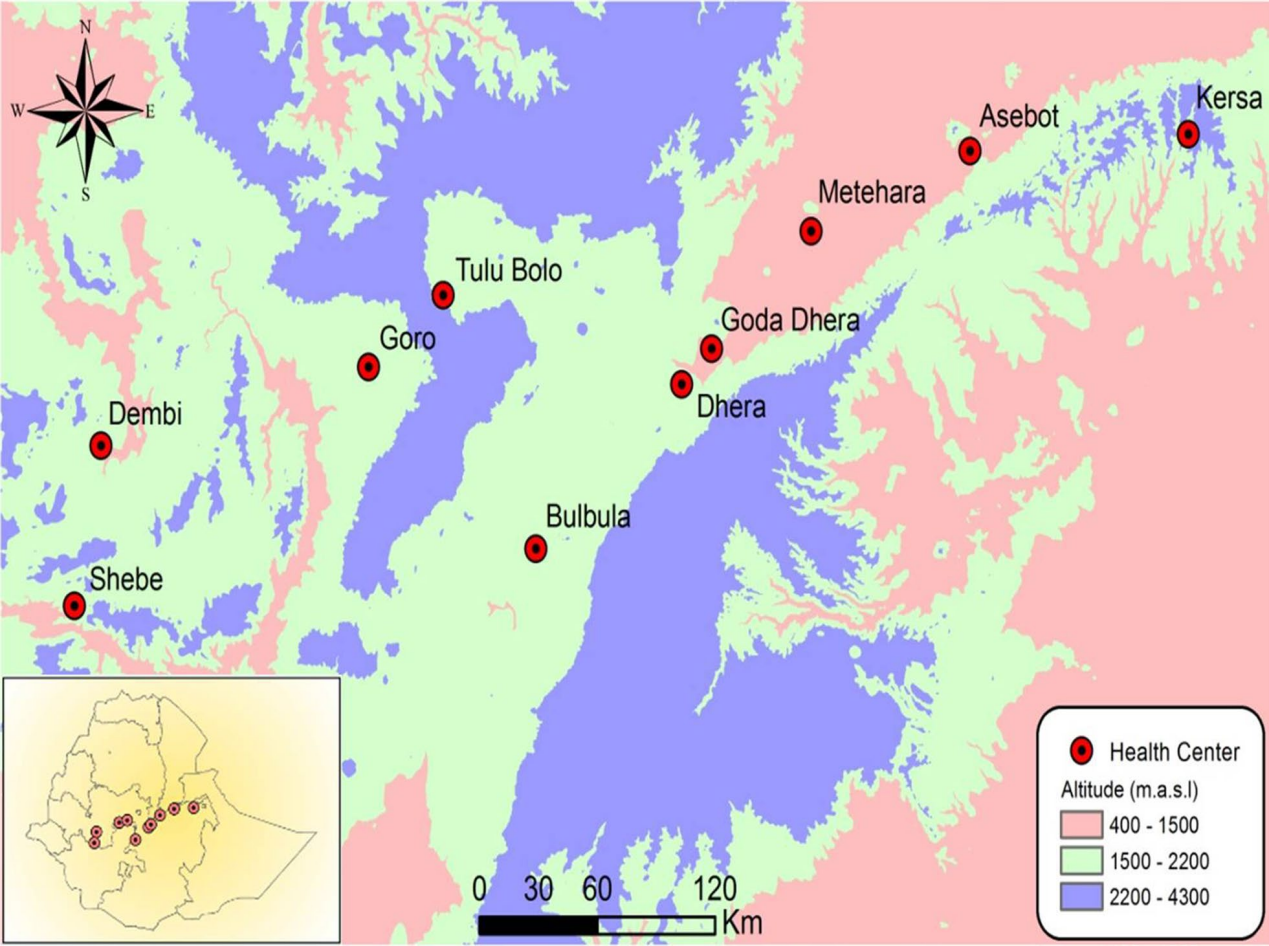
Location of study sites

A community cross sectional study was conducted, in June 2016, mainly in sentinel sites established by the National Malaria Control Programme for malaria surveillance in Ethiopia. Figure 1 shows map of the study area. The sentinel sites were chosen to generate evidence that supports the ongoing malaria control activities and epidemic detection efforts in Ethiopia. The surveillance sites consisted of ten primary health care units (PHCUs) covering a large geographic area, from low to high malaria transmission settings in Ethiopia [16, 17]. Each PHCU serves approximately 25,000 people and consisted of one health centre and five satellite community level health posts. Health centres are usually staffed by trained health professionals and provide full range of services including diagnosis and treatment of cases with uncomplicated malaria, while the health posts are staffed by two salaried health extension workers (HEWs) [18]. At health post level, HEWs conduct diagnosis of malaria using multi-species rapid diagnostic tests (RDT) and provide appropriate treatment accordingly [18]. Eight of the ten sentinel sites were included in the present study and two sentinel sites were replaced (to meet local health system need) by similar primary health care units (Shebe Sombo and Goda Dhera). These sites represent varying degree of malaria transmission settings and wide range of altitude (i.e. ranges from 950 to 2230 m above sea level) [16, 17].

### Population and sample size

The study participants were apparently healthy children 2–9 years of age. Children in this specific age group were chosen considering recommendations by the World Health Organization (WHO) for malaria endemicity study [19, 20]. The sample size was determined using single population formula (n = (Z_1−α/2_)^2^ p (1 − p)/d^2^) based on the following assumptions: Prevalence of malaria (parasite rate) among children (p = 10.5%) [21], 2% marginal error, 95% confidence interval, and 15% non-response rate. This gave a sample size of 1038 children.

### Sampling technique

Children were sampled from ten PHCUs (eight malaria sentinel sites, and two non-malaria sentinel sites from Oromia Regional state, Ethiopia. In brief, sampling process was implemented as follows: First, the sample size (1038 children) was equally allocated to each PHCU. Second, two satellite health posts/villages, which are parts of the PHCU were selected randomly. Equal numbers of children were considered from each of the selected health posts. Within the selected health posts, list of households with eligible children (age 2–9 years) was obtained from family register from health posts. Simple random sampling was employed to select eligible households using the list obtained from health post level. Then, parents were invited to give consent for participation of their children, in the study through local administrators, community volunteers and community-based health workers. Parents were invited to the nearest health facility, (e.g. health centre or health post). Up on arrival, parents/caretakers were given detail information about the study purpose, process and signed written consent for children that participated in the study.

### Malariometric indices

Three basic measures of malariometric indices were used to determine endemicity or malaria transmission which included: spleen rate (SR), parasite rate (PR), and enzyme immunoassay (EIA) malaria antibody test.

### Spleen rate (SR)

This is the proportion of children with palpable enlargement of the spleen. Spleen palpation was conducted by clinicians at the ‘standing position’ technique as suggested by Dempster [20, 22]. The size of enlarged spleen was graded from ‘0’ to “5” according to Hackett’s method of classification of degree of enlarged spleen, where “0” shows normal spleen. Spleen rate was estimated as proportion of children with palpable spleens; individuals of with classes 1–5 of the size of palpated spleen. The average enlarged spleen was determined by multiplying the number of individuals in class except class of zero, by the spleen class number (1–5), summing up these products and divining the total by the number of subjects whose spleens were palpable (grades 1–5).

### Malaria parasitaemia/parasite rate

For detection of malaria parasitaemia and antibody, 3–5 ml venous blood was collected from each child participated in the study. However, for those children who refused venous blood collection or venous blood unavailable, finger prick used to get the specimen for RDT and blood film examinations. For rapid qualitative identification of malaria infections, CareStart malaria Pf/Pv (HRP2/pLDH) Ag Combo RDT (ACCESS BIO, INC., USA) was used. From the collected venous blood, 5 µl whole blood was used for RDT. The RDT test was performed as per the recommendation of the manufacturer [23].

For microscopic examination, thick and thin films were prepared and air dried in the field, and the thin film was fixed with methanol. Both films were stained with 10% Giemsa. Each slide was read by experienced laboratory technologist at Jimma University Specialized Hospital. For positive slides, type of species was reported. In order to ensure the quality of reading, all positives slides and 15% of negatives slides were re-read by another microscopist who was blinded to the initial reading, which resulted in 16.0 and 0.02% discordant readings, respectively. Slides with discordant results were re-read by a third microscopist who was blinded to the previous results.

Absence of malaria parasite in 1000× oil immersion objective in thin blood film was declared negative. A slide was considered as negative after 100 fields were examined and no parasite seen [24]. Both for RDT and microscopy, general parasite rate was defined as the proportion of study subjects (children) harbouring parasites of any species among the sampled children. And species infection rate was defined as the proportions of children harbouring parasites of a given species.

### Enzyme immunoassay (EIA) malaria antibody detection

Malaria EIA test uses four recombinant antigens in a sandwich form and detects antibodies to malaria during all stages of infections. The antigen used was a total extract from *Plasmodium falciparum* cultivation enriched with recombinant antigens from *Plasmodium vivax* (Bio-Rad, France). The antigens detect specific antibodies to the four species of *Plasmodium* parasite species; *P. falciparum, P. vivax, Plasmodium ovale* and *Plasmodium malariae*. Plasma obtained from whole blood collected, from venous of child, into ethylenediaminetetraacetic acid (EDTA) treated was used as specimen for antibody detection. The specimen was stored in − 20 °C freezer, but thawed and well mixed before testing. The test was based on the binding of specific antibodies present in the specimen to antigens immobilized on a 96-well EIA plate. The test was performed as recommended by the manufacturer [25] as follows. 50 µl of specimen (plasma) was added to each antigen coated EIA well plate. In the same plate, 50 µl of positive controls was dispensed into two wells and negatives controls dispensed into three wells; meaning negative control tested three times and positive controls tested twice with each lot of tests. The plate was, then, incubated at 37 °C for 30 min followed by washing each well five times with dilute wash buffer. Then, 50 µl diluted conjugate (i.e. conjugated monoclonal antibodies) solution of horseradish peroxidase was added to all wells and the plate was incubated (covered) again for 30 min at 37 °C before being washed five times. Then, 50 μl of substrate solution (urea peroxide and tetramethyl benzidine) was dispensed into each well and incubated at room temperature for 30 min. Then, 50 μl stop solution (0.5 M sulphuric acid) was added into each well to stop further development of colour. Finally, the plate was read at absorbance wave-length of 450 nm using automated EIA plate reader. The cut-off value was calculated as mean of the optical density (OD) of value of the three negatives control plus 0.100. Samples with an absorbance of 450 nm values greater than cut-off value were considered as positive (presence of specific antibody to *Plasmodium* species) by the malaria EIA antibody test. The test does not distinguish between *Plasmodium* species, nor IgG and IgM and between an acute and chronic infection. The assay carried out at Jimma University Tropical and Infectious Disease Research Centre (TIDRC), using an automated procedure both for washing wells and reading EIA plates.

Based on SR, malaria endemicity is defined as holoendemic (SR > 75%), hyperendemic (51–75%), mesoendemic (11–50%) and hypoendemic (SR < 10%) [20, 22]. The result of malaria EIA antibody test was corroborated with spleen and parasite rates to better understand malaria endemicity in Ethiopia.

In addition to blood sample collections, the background information of each child such as age, sex, body temperature was recorded.

### Statistical analysis

The data were analysed using SPSS version 21.0 and STATA version 12.0. The analysis was done by study sites, altitude and demographic factors. One-way ANOVA and t-test were used to compare mean optical density. Logistic regression was used to assess the association of age, sex and altitude with reactive malaria antibody. Spearman’s correlation was used to assess the correlation among malariometric indices. A 95% confidence interval and p < 0.05 level of significance were used to declare statistically significant association.

## Results

### Background characteristics of studied children

Overall, 763 children participated in this study with response rate of 73.1%. Analysis of splenomegaly rate and parasitaemia was done on all 763 samples. Nevertheless, due to insufficient blood sample, malaria EIA antibody test was done only for 672 samples. More than half (53.1%) the included children were males and the remaining were females. Four hundred forty-one (57.8%) were between 5 and 9 years old and the remaining were between 2 and 4 years of age. In terms of place of residence, 555 (72.7%) were recruited from rural areas.

### Prevalence of malaria parasitaemia

Table 1 presents the prevalence of malaria parasitaemia by RDT and microscopy. The overall prevalence of malaria parasitaemia was 2.49% (95% CI 1.38–3.59) (using RDT) and 2.36% (95% CI 1.28–3.44) by microscopy. *Plasmodium falciparum* was the predominant malaria parasite species, accounting for 62.63% (10/19) of the infections. In microscopy, 11 (57.89%) of the malaria cases were due to *P. falciparum*. No mixed infection was detected by microscopy.

**Table 1:** Prevalence of malaria parasitaemia among children 2–9 years of age, Ethiopia (June 2016)

Parasite species	RDT		Microscopy	
	Overall parasite rate, 95% CI	Infection rate by species, 95% CI	Overall parasite rate, 95% CI	Infection rate by species, 95% CI
*P. falciparum*	1.31% (0.50 to 2.12)	1.57% (0.69 to .46)	1.44% (0.59 to 2.29)	1.44% (0.59 to 2.29)
*P. vivax*	0.92% (0.24 to 1.60)	1.18% (0.41 to 1.95)	0.92% (0.24 to 1.60)	0.92% (0.24 to 1.60)
Mixed infections	0.26% (− 0.10 to 0.63)		0.00%	
Overall	2.49% (1.38 to 3.59)		2.36% (1.28 to 3.44)	

### Malaria parasitaemia by study sites and altitude

Malaria parasitaemia was detected only in three study sites namely Dhera, Metahara and Goda Dhera, in which prevalence of 14.7, 3.6 and 2.3% was documented, respectively. In one of the villages in Dhera catchment (Dirre Kiltu), 33.3% of the sampled children had infection with detectable parasitaemia. Moreover, the prevalence of malaria parasitaemia significantly varied by altitude (x^2^ = 23.255, p = 0.001): higher prevalence was found in areas below 1500 m asl (5.8%) and in areas with mid-altitude (1500–2000 m asl) which was 0.3%. No malaria parasite was detected at an altitude > 2000 m asl. When the analysis included only areas below 2000 m asl, prevalence was 2.70%, by RDT.

### Malaria parasitaemia by age, sex and fever

Only 18 (2.4%) of the surveyed children had fever (axillary body temperature ≥ 37.5 °C) at the time of the study. Fever was less correlated with detectable parasitaemia (r = 0.085, p = 0.019). Of those positive children by RDT, only 4 (21.1%) were febrile. The difference in prevalence of malaria parasitaemia between males (2.7%) and females (2.2%) and children ≥ 5 and those below 5 years of age (2.9% vs 1.9%) was not statistically significant (p > 0.05).

### Splenomegaly rate

The prevalence of enlarged spleen was 1.70% (95% CI 0.78–0.2.66%), with mean enlarged spleen 1.85 (SD = 0.38). Enlarged spleen was detected in only five of the study sites. Relatively higher rates of splenomegaly were recorded in Dhera (5.3%), Goda Dhera (4.6%) and Goro (3.3%) sites. Rate of splenomegaly was higher among children living in areas < 1500 m asl (2.9%) and among children who resided in areas > 2000 m asl (1.4%). In areas between 1500 and 2000 m asl, splenomegaly was seen in only 0.8% of the children. Furthermore, splenomegaly was higher among children greater ≥ 5 years of age (2.3%) as compared to children < 5 years of age (0.9%). Similarly, the rate was slightly higher among females (2.0% females vs 1.5% males). Yet, none of these differences was statistically significant (p > 0.05).

### Acquired anti-malarial antibody

**Fig. 2:**
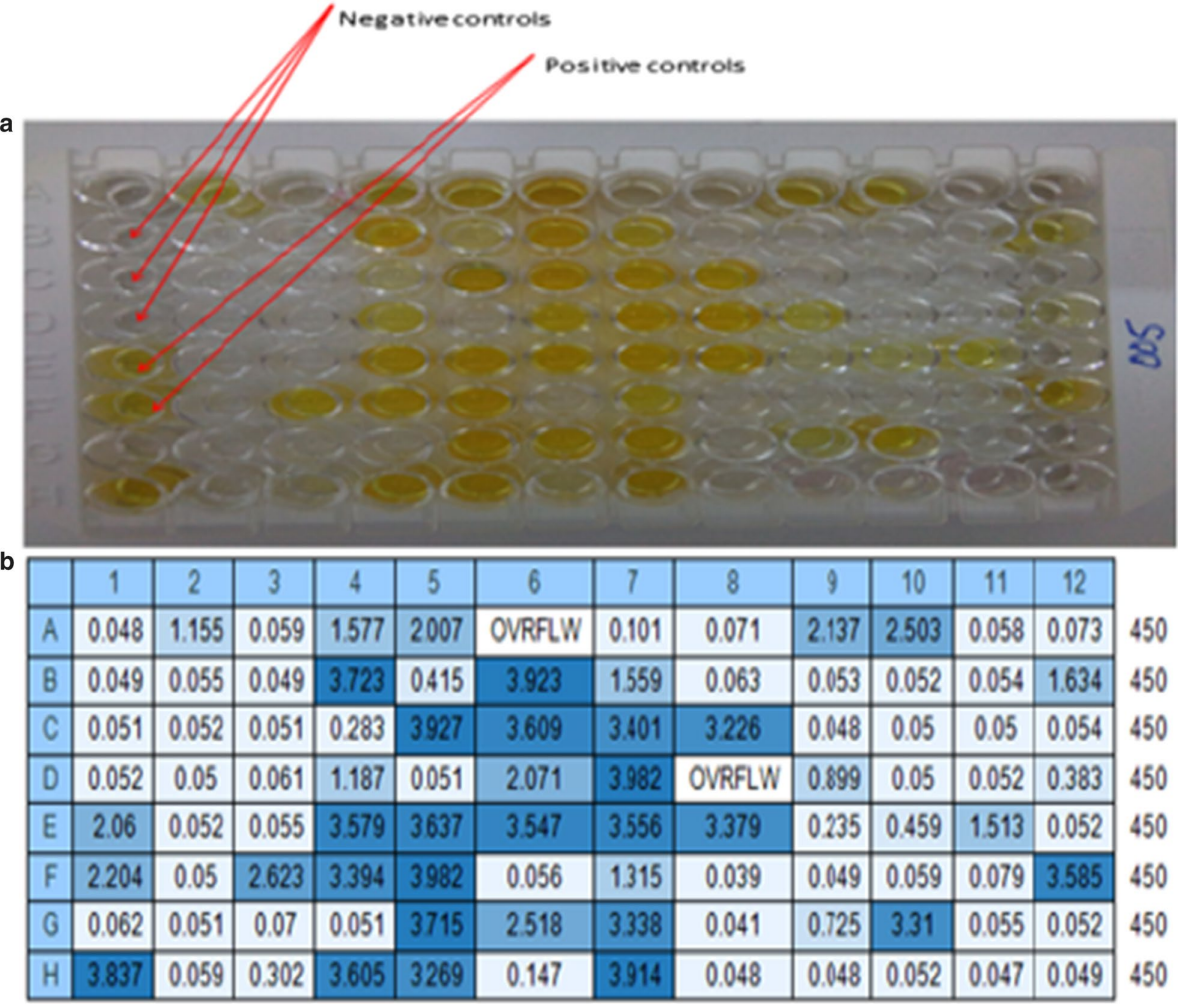
An image of malaria EIA 96 test plate, June 2016, Ethiopia. **a** Triplicate negative controls and two positive controls. The yellow colour indicates reactive samples to recombinant malaria antigens in a sandwich EIA test. The higher the intensity of the yellow colour, higher intensity of reactive anti-malarial antibody. **b** Indicates optical density (OD) reading of the EIA plate with an absorbance of 450 nm values. The higher OD reading and the stronger the positive anti-malarial antibody

**Fig. 3:**
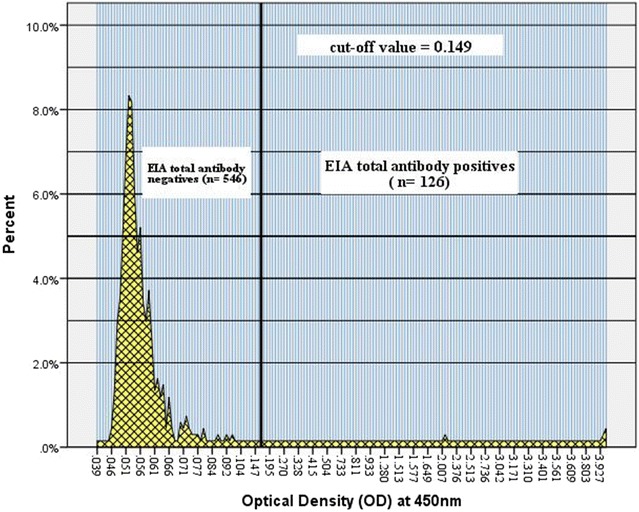
Distribution of total malaria antibody optical density readings at an absorbance reading of 450 nm for the whole sample, Ethiopia, June 2016. The mean OD was 0.42 (SD = 0.94) for the whole sample. The distribution was skewed to the left indicating that the absorbance value was low for large percentage of the sample

**Fig. 4:**
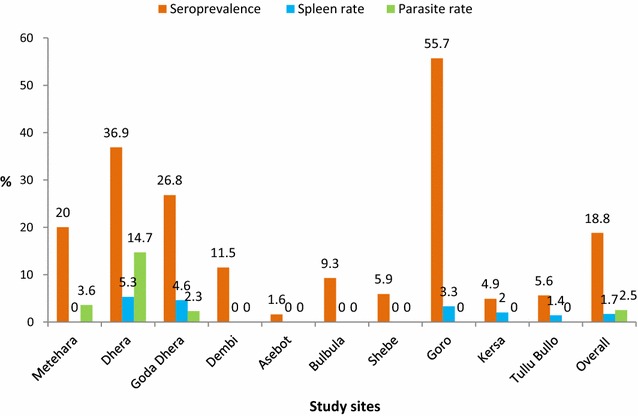
Malariometric indices by study sites, Ethiopia, June 2016. As indicated in this figure, acquired immunity was significantly variable by study sites. The highest rate of acquired immunity for malaria was observed in three study areas, namely: Goro (55.7%) followed by Dhera (36.9%) and Goda Dhera (26.8%). Lower prevalence of reactive malaria antibody was observed in Asebot (1.6%), Kersa (4.9%), Tullu Bollo (5.6%) and Shebe (5.9%)

**Fig. 5:**
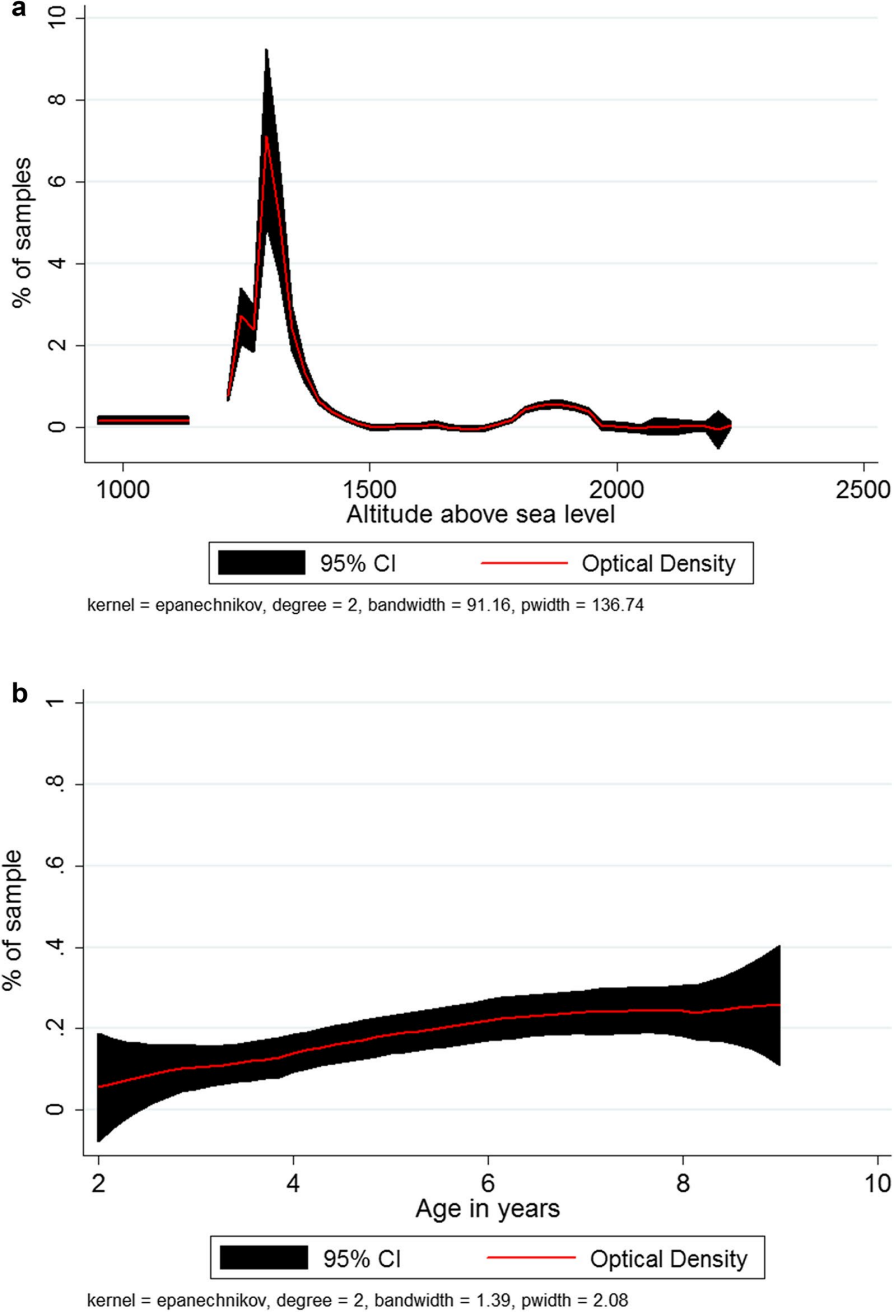
Acquired total malaria antibody among children 2–9 years old by altitude (**a**) and age (**b**), Ethiopia, June 2016. This figure shows that seropositive rate had negative correlation with altitude, but positive correlation with age. Malaria antibody reactivity decreased as altitude increased (**a**) but increased as age increased (**b**)

Figure 2 depicts an image of one of the malaria EIA tests. The distribution of OD reading is indicated in Fig. 3. Overall, 126 (18.75%; 95% CI 15.79–21.71) of the children were positive for total malaria antibody. The prevalence of malariometric indices, by study site, is shown in Fig. 4. The acquired anti-malaria immunity was greatly influenced by age, sex and altitude (Table 2). Seropositivity rate for children under the age of five was lower by 49.0% as compared to children ≥ years of age. Likewise, children living in areas < 1500 m asl were 5.86 times more likely to have reactive malaria antibody as compared to children residing in areas > 2000 m asl (OR = 5.86, 95% CI 2.05–16.70, p = 0.001). The level of acquired immunity was low (5.6%) in areas with > 2000 m asl. A close look into level of exposure by specific localities/villages depicted heterogeneous malaria transmission that ranged from zero to 80.0% (Dirre Kiltu, Dhera site). Figure 5 shows acquired immunity to malaria by altitude (a) and age (b).

**Table 2:** Association of age, sex and altitude with malaria seropositive, Ethiopia (June 2016)

Variables	Prevalence of malaria antibody (%)	p-value	OR	95% CI
Sex of child				
Male	20.9	0.212	1.29	0.86–1.93
Female	16.3			1
Altitude above sea level (m)				
< 1500	25.8	0.001	5.86	2.05–16.70
1500–200	15.5	0.044	2.97	1.03–8.53
> 2000	5.6			1
Age of child (years)				
< 5	13.1	0.003	0.51	0.33–0.79
≥ 5	22.1			1

### Relationship between accesses to LLIN exposure to malaria

In this study, ownership, access to and use of LLIN did not have significant association with exposure to malaria (p > 0.05). The prevalence of reactive antibody was 19.8 and 19.5% among households who own any nets and those without nets, respectively. Similarly, the prevalence of reactive antibody was 21.3 and 16.4% among children who slept under LLIN and those who did not, respectively. Likewise, rate of exposure to malaria did not vary by extent of access to LLINs: seropositive rate was 19.5, 17.9 and 21.3% among households with no access, sufficient access (i.e. 2 nets for every two people in the household) and insufficient access, respectively.

### Intensity of acquired malaria antibody

**Fig. 6:**
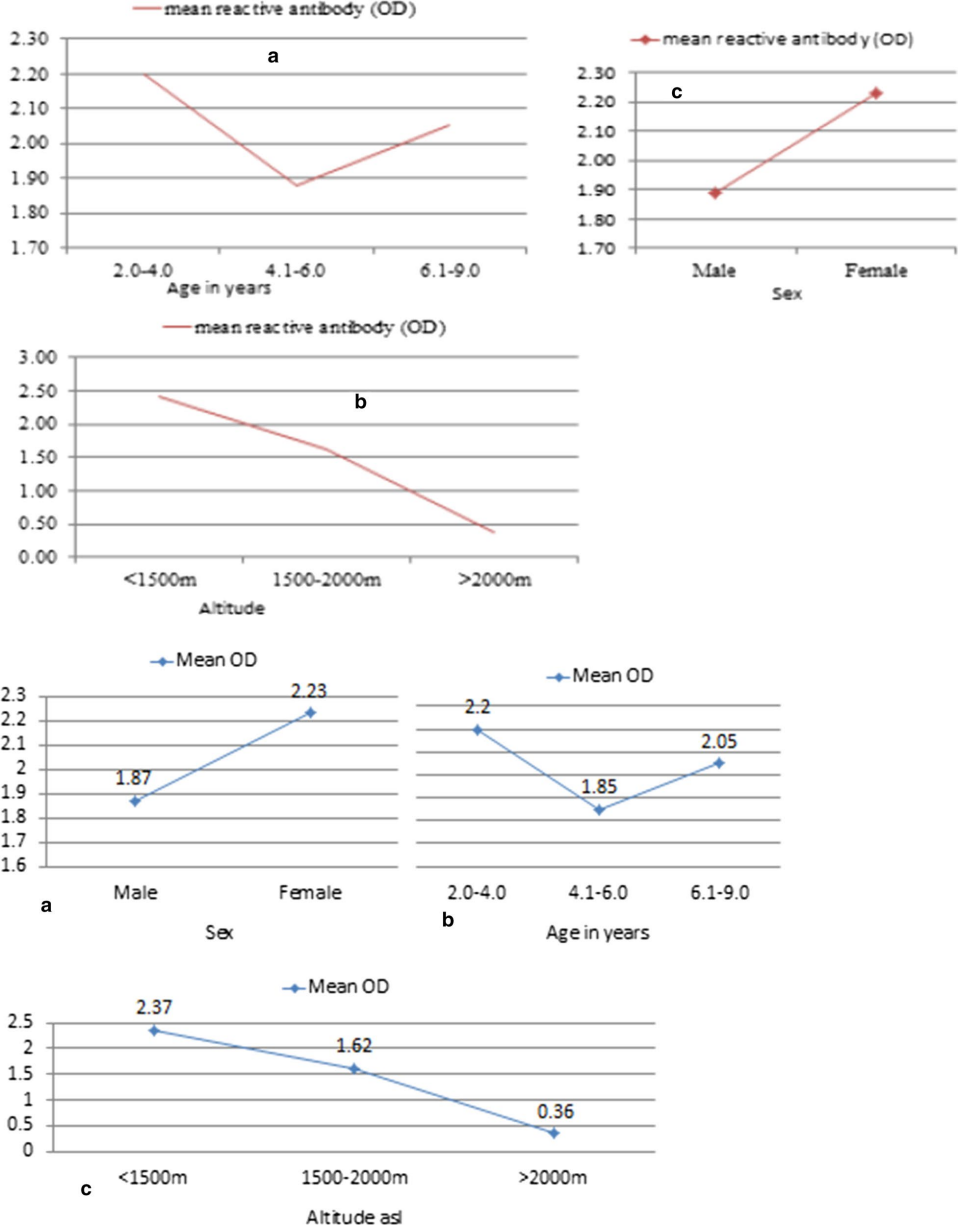
Mean OD readings of malaria antibody by **a** age, **b** sex and **c** altitude among positively reacted samples, Ethiopia (June 2016). The strength of malaria antibody was feeblest among children 4–6 years old, but highest for children 2–4 years. On the other hand, as altitude increased, the intensity of malaria antibody decreased (**b**). Males had weaker malaria antibody even when positively reacted to EIA antibody test (**c**)

The mean OD among malaria antibody positive samples was 2.01 (SD = 1.29). Figure 6 shows mean OD by age (a), altitude (b) and sex (c).

### Comparison of malariometric indices and endemicity level

Of the samples tested, reactive by malaria EIA (n = 126), 14.3% (18/126) and 13.5% (17/126) had detectable parasitaemia by RDT and microscopy, respectively. Table 3 presents comparison of malariometric indices. Of positive samples using RDT and microscopy, 94.7% (n = 19) and 94.4%, were reactive to malaria EIA antibody test, respectively. Only 53.8% of the children with enlarged spleen had reactive malaria antibody. On the other hand, only 10.3% (13/126) of the children with reactive antibody had palpable spleen.

**Table 3:** Comparison among malariometric indices and their correlation, Ethiopia (June 2016)

Malariometric indices	Malaria EIA	
	Positive, n (%)	Negative, n (%)
Parasitaemia (RDT)		
Positive	18 (94.7)	1 (5.3)
Negative	108 (16.5)	545 (83.5)
Parasitaemia (microscopy)		
Positive	17 (94.4)	1 (5.6)
Negative	109 (16.7)	545 (83.3)
Splenomegaly		
Positive	7 (53.8)	6 (46.2)
Negative	119 (18.1)	540 (81.9)
Reported exposure to malaria		
Positive	81 (39.1)	126 (60.9)
Negative	41 (10.0)	369 (90.0)

The association between different measures of malariometric indices was weak (Table 4). Splenomegaly was weakly correlated with acquired immunity (r = 0.13, p < 0.001) and parasitaemia (r = 0.18, p < 0.05). The highest correlation was recorded between parasitaemia and acquired immunity (r = 0.33, p < 0.001). Based on splenomegaly rate (1.7%), the overall malaria endemicity can be classified as *‘Hypoendemic’*. However, malaria antibody assay showed a higher level of cumulative exposure (18.75%) to malaria of the target communities with greater degree of heterogeneous and localized transmissions.

**Table 4:** Correlation among malariometric indices, Ethiopia, June 2016

Indices	Malaria EIA	Parasitaemia (RDT)	Parasitaemia (microscopy)	Splenomegaly	History of malaria (reported)
Malaria EIA	1.00				
Parasitaemia (RDT)	0.33**	1.00			
Parasitaemia (microscopy)	0.32**	0.97**	1.00		
Splenomegaly	0.13**	0.17**	0.18**	1.00	
History of malaria (reported)	0.35**	0.16**	0.16**	0.08*	1.00

** p < 0.0, * p < 0.05

## Discussion

This study investigated the level of malaria transmission in Ethiopia based on classical malariometric indices (parasitaemia and spleen rate) and EIA for the detection of specific antibodies to *Plasmodium* species. The overall prevalence of malaria parasitaemia was 2.5% (using RDT) although higher heterogeneity was observed among localities and with different altitude. The finding of this study was higher than many community-based reports in Ethiopia, including the 2007, 2011 and 2015 malaria indicator surveys (MIS) [26–30], but quite low as compared to parasite prevalence rate from some African countries [31, 32]. Congruent with MIS data in Ethiopia [26–28], high rate of infections was recorded in areas below 1500 m asl. Consistent with previous studies in Ethiopia [12, 13, 17, 18, 26–30, 33, 34], *P. falciparum* was the predominant malaria parasite species in the study population. Moreover, the declining trend of malaria burden was less prominent in *P. falciparum* and there was an indication of resurgence of *P. falciparum* in 2010 and 2013 in the country [17]. Generally, the prevalence of *P. falciparum* and *P. vivax* varies according to locality and seasons, in Ethiopia [21, 26–30, 33]. Yet, the persistence higher prevalence of *P. falciparum* conveys important messages as it is the primary target for malaria control and elimination programme [35].

Splenomegaly has been used as stable indicator of malaria endemicity for many decades [20]. In this study, overall malaria seemed to be hypoendemic in all the study sites with consistently low level of splenomegaly across study sites, even zero in some settings. In hypoendemic situation, malaria transmission is seasonal or intermittent following unusual rainfall with minimal impact on community health [20]. Nevertheless, the impact of malaria is still significant in Ethiopia [12, 13]. The reliability of splenomegaly as a proxy for levels of malaria transmission can be affected by several factors. In situations of acute infection and unstable transmission, where community members lacks extend exposure, splenomegaly can be close to zero [19, 36]. Of course, despite history of high malaria transmission in some study sites (e.g. Bulbula and Tullu Bollo) [17], the rate of splenomegaly was quite low and even zero in Bulbula site. Splenomegaly is a useful measure of malaria risk mostly in areas with stable malaria transmission [19]; hence, it is not a good indicator of malaria endemicity for Ethiopia.

Essentially, each individual develops antibodies to *Plasmodium* species following infection [37, 38]. Thus, the presence of malaria antibodies in the human serum or plasma is a marker of recent exposure to malaria. In this study, EIA demonstrated that the magnitude of exposure to malaria was much higher (as compared to splenomegaly and parasitaemia) among the study population, with higher heterogeneity by altitude and localities. Except one school-based study with seroprevalence rate of < 12.0% [39], there is no published data comparing acquired immunity to malaria in Ethiopia. The present serological study revealed that malaria transmission was marginal among populations living in areas > 2000 m asl in contrast to areas below 1500 m asl which were characterized by relatively high malaria transmission. Furthermore, the heterogeneity of transmission was quite high among study villages. This suggests that malaria EIA antibody test can serve as a good proxy of the relative risk of infection or levels of malaria transmission, particularly in low transmission settings. In low transmission settings, the level of circulating parasitaemias often falls below a detectable threshold, and in such context, neither splenomegaly nor parasitaemia provide realistic information both on the heterogeneity and levels of the transmission [5–11]. The Ethiopian 2015 MIS report also realized that the existing MIS measures were not able to distinguish malaria risk areas; and hence it is not a proper tool for stratification of malaria risk in Ethiopia [28]. Therefore, malaria EIA assay that uses four recombinant antigens can serves as a useful method to assess localized risk and levels of malaria transmissions which could be useful to guide elimination initiatives in Ethiopia and in other similar settings.

Even though stratification of malaria endemicity on the basis of serological tests not common; generally, malaria in this study appeared to be *mesoendemic* considering partial immunity as manifestation of chronic malaria. According to this class of endemicity, malaria is typically characterized by regular seasonal transmission under normal rainfall conditions [19, 20, 22]. However, to be precise, no single class of endemicity provides a clear picture of malaria transmission in the present context. Instead, low and spatially heterogeneous transmission was documented representing the dynamics of malaria transmission-involving multiple forms of endemicity with highly localized and clustered transmissions supporting ongoing pocket of local transmission. The fact that the rate of exposure to malaria reaches 80% in some of the study villages proves the nature and extent of heterogeneity. Therefore, malaria control and elimination programmes, in Ethiopia, need to understand the local malaria transmission to deploy tailored malaria resources and enhance elimination efforts. Targeted and location-specific interventions with strong surveillance and responsive actions especially in hardest to reach areas where pockets of active/ongoing transmissions are most likely (e.g. Dhera) need to be carefully designed for successful control and ending malaria transmission. In low altitude areas, communities who reside near water bodies such as in development project and corridors that serve as potential mosquito breeding grounds need priority in targeting malaria interventions. Unless these entire potential localized sources are identified and addressed, elimination initiatives may remain unattainable. On the other hand, in areas where malaria transmission has declined, it is essential to maintain community adherence to malaria preventive measures as people may become increasingly less concerned about malaria [2, 40].

Although chronic exposure to malaria was higher among children over the age of 5 years and among males, a high level of circulating antibodies was detected among younger children and females. It is not quite clear why younger children and females demonstrated stronger anti-malarial antibodies. Maybe, the intensity of antibodies varies with durations of exposure, and clinical and immunological response may also vary between males and females. This needs further investigation.

Information on the level of herd immunity to malaria has several practical implications. High levels of immunity in the population could help to accelerate elimination success by reducing transmission rate [41]. The level of acquired immunity to malaria was pretty low in the present settings implying low transmission context which is easier to initiate and take the elimination process forward. On the contrary, low immunity means that a large percentage of the population is at increased risk with major consequences; even risk of major epidemics can be higher if control measures fail to be sustained [35, 41]. Thus, settings with low level of acquired immunity to malaria should be constantly monitored to reduce these potential risks. Moreover, people with partially protective immunity can serve as reservoir of malaria infection in the community [5, 6, 9–11].

In this study, no parasitaemia was detected among the majority of the febrile children; children with high intensity antibodies. This creates a critical challenge to malaria elimination programme since many people who are infected with malaria parasites remain undetected by the healthcare system [2, 5, 6, 9–11]. This study also showed that access and use of LLINs did not provide significant protective effect on exposure to malaria. This may suggest that most infections were due to residual transmission. Of course, there is an urgent need to evaluate the impact of LLINs distribution and utilization on malaria-antibody response particularly in settings that recently experienced significant malaria reduction.

### Strength and limitations of the study

The major strength of this study was the use of a combination of classical and immunological technique through community based approach; involving multiple sites with relatively large sample size. The finding of the study can be generalized to other similar malaria endemic settings. This is the first report from Ethiopia demonstrating serological tests to measure levels of malaria transmission and distribution of partially protective malaria anybody in the population. Thus, the finding can serve as first-hand information in planning malaria elimination. However, lower response rate might have affected reliability of estimates.

## Conclusions

The prevalence of malaria parasitaemia was somewhat high compared to many earlier studies, with high prevalence of *P. falciparum*. The classical methods used in measuring malaria endemicity, namely, rate of splenomegaly and parasite rate failed to reflect the level and heterogeneity of malaria risk in such low transmission settings. In contrast, EIA malaria antibody test was able to detect heterogeneity of levels of malaria transmission and provided more precise and clear picture of variations in levels of transmission in the Ethiopian context. The levels of transmission were quite local and heterogeneous by altitude and local ecology, manifesting multiple endemicity levels. Areas with low altitude carry the highest burden of malaria transmission whereas malaria transmission in high attitude settings was minimal. Therefore, in-depth understanding of the local level ongoing risk of transmission should be an initial step for countries with low transmission and different disease eco-epidemiology initiating elimination programme. The study also suggests the need to incorporate serological test into large scale periodic malaria surveys such as malaria indicators surveys, particularly in areas with low transmission and where elimination programmes are initiated. Serological test need to be considered in addition to data which is often extracted from existing health facilities, records of malaria control programmes and a point in time data from malaria indicator survey (MIS). Malaria elimination initiatives need to adopt location-specific strategies and tailor its approach to ongoing and local specific malaria transmission.

## Abbreviations

EIA: enzyme-linked immunosorbent assay
HEWs: Health Extension Workers
ITNs: insecticide-treated nets
MDG: millennium development goal
OD: optical density
RDT: rapid diagnostic test
SR: parasite rate
PHCUs: Primary Health Care Units
SR: spleen rate
WHO: World Health Organization
